# AI-Powered Segmentation of Invasive Carcinoma Regions in Breast Cancer Immunohistochemical Whole-Slide Images

**DOI:** 10.3390/cancers16010167

**Published:** 2023-12-29

**Authors:** Yiqing Liu, Tiantian Zhen, Yuqiu Fu, Yizhi Wang, Yonghong He, Anjia Han, Huijuan Shi

**Affiliations:** 1 Institute of Biopharmaceutical and Health Engineering, Tsinghua Shenzhen International Graduate School, Shenzhen 518055, China; liuyiqin20@mails.tsinghua.edu.cn (Y.L.); fu-yq22@mails.tsinghua.edu.cn (Y.F.); yz-wang22@mails.tsinghua.edu.cn (Y.W.); heyh@sz.tsinghua.edu.cn (Y.H.); 2 Department of Pathology, The First Affiliated Hospital of Sun Yat-sen University, Guangzhou 510080, China; zhentt3@mail.sysu.edu.cn

**Keywords:** artificial intelligence, breast cancer, IHC quantification, invasive carcinoma, Ki-67

## Abstract

**Simple Summary:**

This study proposes an innovative approach to automatically identify invasive carcinoma regions in breast cancer immunohistochemistry whole-slide images, which is crucial for fully automated immunohistochemistry quantification. The proposed method leverages a neural network that combines multi-scale morphological features with boundary features, enabling precise segmentation of invasive carcinoma regions without the need for additional staining slides. The model demonstrated an impressive intersection over union score on the test set, and a fully automated Ki-67 scoring system based on the model’s predictions exhibited high consistency with the scores given by experienced pathologists. The proposed method brings the breast cancer fully immunohistochemistry quantitative scoring system one step closer to clinical application.

**Abstract:**

Aims: The automation of quantitative evaluation for breast immunohistochemistry (IHC) plays a crucial role in reducing the workload of pathologists and enhancing the objectivity of diagnoses. However, current methods face challenges in achieving fully automated immunohistochemistry quantification due to the complexity of segmenting the tumor area into distinct ductal carcinoma in situ (DCIS) and invasive carcinoma (IC) regions. Moreover, the quantitative analysis of immunohistochemistry requires a specific focus on invasive carcinoma regions. Methods and Results: In this study, we propose an innovative approach to automatically identify invasive carcinoma regions in breast cancer immunohistochemistry whole-slide images (WSIs). Our method leverages a neural network that combines multi-scale morphological features with boundary features, enabling precise segmentation of invasive carcinoma regions without the need for additional H&E and P63 staining slides. In addition, we introduced an advanced semi-supervised learning algorithm, allowing efficient training of the model using unlabeled data. To evaluate the effectiveness of our approach, we constructed a dataset consisting of 618 IHC-stained WSIs from 170 cases, including four types of staining (ER, PR, HER2, and Ki-67). Notably, the model demonstrated an impressive intersection over union (IoU) score exceeding 80% on the test set. Furthermore, to ascertain the practical utility of our model in IHC quantitative evaluation, we constructed a fully automated Ki-67 scoring system based on the model’s predictions. Comparative experiments convincingly demonstrated that our system exhibited high consistency with the scores given by experienced pathologists. Conclusions: Our developed model excels in accurately distinguishing between DCIS and invasive carcinoma regions in breast cancer immunohistochemistry WSIs. This method paves the way for a clinically available, fully automated immunohistochemistry quantitative scoring system.

## 1. Introduction

Breast cancer is the most prevalent cancer worldwide, with a high mortality rate [1]. Invasive breast carcinoma of no special type (IBC-NST) is the common histological subtype of breast cancer [2]. Subdividing breast cancer into different molecular subtypes is crucial for guiding treatment decisions. The main molecular subtypes of breast cancer include luminal A, luminal B, human epidermal growth factor receptor 2 (HER2) enriched, and basal-like [3].

Immunohistochemistry (IHC) analysis is a cost-effective and widely used method for molecular subtyping [4,5]. It involves analyzing the status of estrogen receptor (ER), progesterone receptor (PR), HER2, and Ki-67. This analysis is performed by applying specific antibodies to tumor tissue samples and observing their reactions under a microscope. Pathologists evaluate the antibody reactions to determine the molecular subtype of the tumor [6]. Quantification of ER, PR, and Ki-67 requires assessing the number of immune-negative and immune-positive tumor cells in a given area [7]. The evaluation of HER2 status is based on cell membrane immunostaining intensity, integrity, and the percentage of membrane-positive cells [8].

Traditionally, immunohistochemical diagnosis relies on manual examination by trained pathologists. IHC slides for the four biomolecules are prepared separately and analyzed using microscopes to estimate biomolecule expression. This process involves visually evaluating hundreds of cells in all invasive carcinoma areas, making it tedious, error-prone, and observer-dependent [9,10,11]. Such factors reduce the significance of histopathological patterns in guiding treatment decisions. Additionally, in low- and middle-income countries, pathology and laboratory services, as well as experienced pathologists, are scarce. This labor-intensive procedure becomes even more expensive and time-consuming in these regions.

Whole-slide imaging (WSI) technology offers a solution to these challenges. Digital pathology allows high-resolution digital imaging of IHC slides, enabling pathologists to examine them on computers, reducing the burden of using microscopes [12,13,14]. Deep learning, a branch of artificial intelligence, has shown significant progress in pathological image analysis [15,16,17,18]. It automatically extracts representative features and underlying information from raw data. Applying deep learning models to the automated analysis of digital pathological sections can further reduce the workload of pathologists and improve consistency in clinical diagnostic practice [19]. An AI-assisted pathological diagnosis system can significantly enhance the speed, accuracy, and objectivity of immunohistochemical quantitative diagnostics.

Several studies have focused on immunohistochemical quantification [20,21,22,23,24,25,26,27], demonstrating high consistency with reference scores and the feasibility of computer-assisted immunohistochemical scoring. However, existing algorithms have limitations that hinder their clinical application. Notably, most methods have not adequately addressed the exclusion of DCIS regions in WSIs. Although some studies have considered this [24], the selection process is still manual, requiring pathologists to manually identify the invasive carcinoma regions in the clinical application stage. Therefore, full automation has yet to be achieved.

For WSI-level invasive carcinoma region segmentation models, some progress has been made in segmenting invasive carcinoma regions in H&E-stained WSIs and distinguishing DCIS from invasive carcinoma [28,29,30]. However, direct segmentation of invasive carcinoma regions in IHC-stained WSIs remains a challenging task. The differential features of invasive and in situ carcinoma in immunohistochemistry images are less apparent, making judgment more difficult. Even experienced pathologists sometimes rely on H&E and special-stained slides to distinguish invasive and in situ carcinoma in IHC-stained slides.

**Figure 1:**
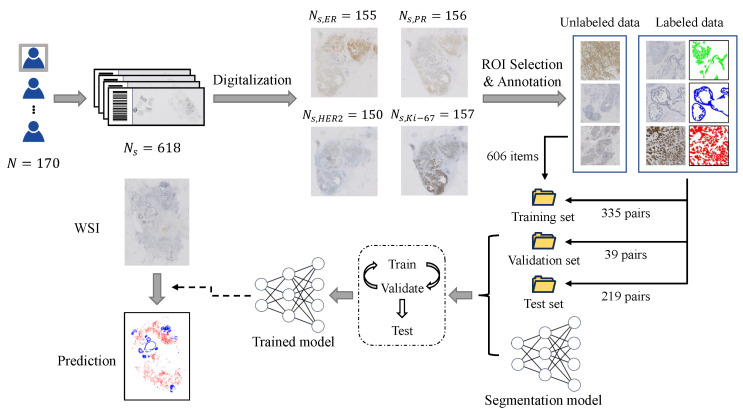
Overview of the proposed approach for invasive carcinoma segmentation in breast cancer IHC-stained WSIs. The methodology includes the following steps: (1) Creation of an epithelial tissue segmentation dataset; (2) Training the segmentation model; (3) Thorough evaluation of the model’s segmentation performance on the test set; (4) Selection of the best-performing model for the application of new WSI inference.

In this study, we have developed a novel approach to segment invasive carcinoma regions in breast cancer IHC-stained WSIs. To achieve this, we first created an epithelial tissue segmentation dataset and trained the initial segmentation model using semi-supervised learning techniques. Subsequently, we enhanced the model by incorporating a multi-scale fusion mechanism and fine-tuning the fusion modules, enabling it to effectively utilize contextual information and deliver more precise segmentation outcomes. The model’s segmentation performance was thoroughly evaluated on a test dataset, and the best-performing model was selected for the application of new WSI inference. The research overview is depicted in Figure 1. Additionally, we assessed the feasibility of the model for clinical-level immunohistochemical quantitative scoring, as outlined in Section 3.3.

## 2. Method

### 2.1. Dataset Construction

We retrospectively selected 170 patients with IBC-NST who underwent IHC examination from January 2022 to January 2023 at Sun Yat-sen University Affiliated First Hospital. The immunohistochemical slides of patients, including ER, PR, HER2, and Ki-67, were obtained. The slides were scanned using an SQS-600P scanner (0.09 μm/pixel). After excluding missing and poor-quality slides, there were various numbers of ER, PR, HER2, and Ki-67-stained slides left. These slides will be used for model training, validation, and testing. We also scanned H&E-stained slides from all patients, and P63 -tained and Calponin-stained slides from some patients for validation. This study was approved by the institutional review board of Sun Yat-sen University Affiliated First Hospital. Due to the retrospective nature of this study, patient-informed consent was not required.

We selected 100 cases from the 170 cases for training of the segmentation model, and the remaining 70 cases for model testing. Subsequently, we selected 980 ROIs from 399 WSI from cases used for training, and selected 374 out of these regions of interest (ROIs) for labeling. Then, we randomly divided these labeled ROIs into a 9:1 ratio for model training and validation. We selected 219 ROIs from 219 WSIs from cases used for testing. The sizes of the ROIs ranged from 2000 to 20,000, and they could be classified into four categories according to the type of epithelial tissue in the image: normal (all epithelial tissue belonged to normal ducts and lobules), in situ carcinoma, invasive carcinoma, and mixed type (epithelial tissue from at least two different types of tissue).

We annotated the contours of the epithelial tissue regions for all ROIs of the first three types and some ROIs of the last type. The annotation was performed using the open-source software QuPath v0.4.3 [31]. For the unlabeled data in the remaining ROIs of the last type (all from training cases), we employed a semi-supervised learning algorithm (see Section 2.2.2 for details) to enable the model to learn from this unlabeled data. This approach alleviates some of the annotation workload and enhances the robustness of the model.

In addition, to rapidly annotate the contour masks of the tumor regions, we adopted a semi-automatic annotation method similar to that used in [14]. First, we annotated some ROIs (100) in the dataset and used them to train a segmentation model. The model only segmented the epithelial tissue without distinguishing its specific type. For the remaining ROIs in the dataset, we first used this pre-trained semantic segmentation model to perform pre-segmentation of the epithelial tissue region, and then modified it based on the pre-segmented contour to greatly accelerate the annotation speed.

All immunohistochemical scores from the test set were obtained from the pathological reports and reviewed by a senior pathology expert.

### 2.2. Training Framework

**Figure 2:**
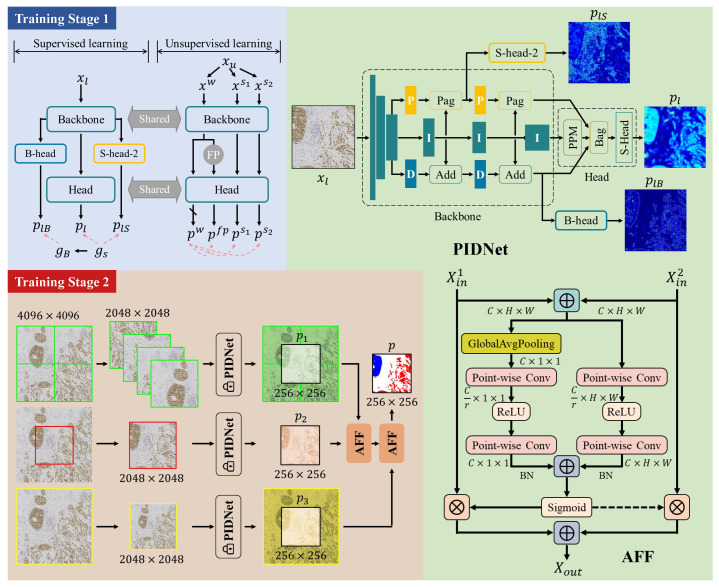
Two-stage multi-scale segmentation model training framework. During Training Stage 1, we utilize semi-supervised learning to train the initial segmentation model. In Training Stage 2, the main focus is on training the multi-scale fusion modules.

This section presents our novel two-stage multi-scale segmentation model training framework designed for segmenting invasive carcinoma regions in breast cancer immunohistochemistry images, as depicted in Figure 2. Firstly, we introduce our initial segmentation model, followed by a detailed description of Training Stage 1, which utilizes semi-supervised learning to train the initial segmentation model. Finally, we delve into Training Stage 2, which primarily focuses on the training of the multi-scale fusion modules.

#### 2.2.1. Initial Segmentation Model

We use PIDNet [32] as our initial segmentation model. We use this model for two main reasons. First, the PIDNet family achieves the best trade-off between inference speed and accuracy, with testing accuracy superior to all existing models. Second, the model introduces an auxiliary derivative branch (ADB) and incorporates boundary attention to guide the fusion of detailed and context branches with ADB’s boundary detection capabilities. This design is well suited for distinguishing in situ carcinoma from invasive carcinoma because the two are almost identical in internal features, and boundary features are key to distinguishing them.

PIDNet uses cascaded residuals as the backbone to achieve a hardware-friendly architecture, which is shown in Figure 2. It has three branches with complementary responsibilities, which are (i) Proportion (P) branch: parse and preserve detailed information in its high-resolution feature map; (ii) Integration (I) branch: locally and globally aggregate contextual information to parse long-range dependencies; and (iii) Differentiation (D) branch: extract high-frequency features to predict boundary regions.

The three branches of PIDNet are inherently complementary, and they use boundary attention in the final stage to guide the fusion of the detailed and context branches. Furthermore, for an efficient implementation, we have set the depths of the P, I, and D branches to medium, deep, and shallow, respectively. Consequently, by varying the depth and width of the model, we have created a family of PIDNet models, namely PIDNet-S, M, and L. In this study, we use the medium-sized PIDNet model. A detailed introduction to the PIDNet can be found in the original paper [32].

During the training stage, the images in the training set are randomly scaled between 0.5 and 1.5 magnification. These scaled images are then randomly cropped into 1024×1024-sized images. Subsequently, data augmentation techniques such as random flips, color jittering, and color normalization are applied to augment the training data before feeding it into the network. The model generates three outputs: $p_{lS}$, $p_{l}$, and $p_{lB}$, which are utilized for calculating the loss function in the subsequent steps.

The loss functions for the P branch and D branch are denoted as $l_{0}$ and $l_{1}$, respectively. $l_{0}$ uses weighted cross-entropy loss to encourage $p_{lS}$ to approach the semantic segmentation ground truth $g_{s}$, while $l_{1}$ utilizes weighted binary cross-entropy loss to improve the fitting of $p_{lB}$ to the boundary ground truth $g_{B}$. To regulate the output $p_{l}$ of the I branch, we employ two loss functions: $l_{2}$, a weighted cross-entropy loss, and $l_{3}$, the boundary-aware CE loss (BAS-Loss [33]). The computations for BAS-Loss are as follows:

(1) $$l_{3}=-\sum_{i,c}\left\{1:b_{i}>t\right\}\left(s_{i,c}\log\hat{s_{i,c}}\right),$$

where *t* is a predefined threshold, and $b_{i}$, $s_{i,c}$, and $\hat{s_{i,c}}$ represent the output of the boundary head for class *c*, the ground-truth segmentation output, and the predicted result of the *i*-th pixel, respectively. Thus, the supervised loss $L_{s}$ can be represented as:

(2) $$L_{s}=\lambda_{0}l_{0}+\lambda_{1}l_{1}+\lambda_{2}l_{2}+\lambda_{3}l_{3},$$

where $\lambda_{0}$, $\lambda_{1}$, $\lambda_{2}$, and $\lambda_{3}$ are weight coefficients, and their values are determined based on empirical observations from experiments.

#### 2.2.2. Training Stage 1: Semi-Supervised Learning

To better utilize the unlabeled data in the training set and train the model effectively, we adopt the semi-supervised semantic segmentation algorithm Unimatch [34]. This algorithm is grounded on the principle of consistency learning, wherein the objective is to maintain output consistency for unlabeled images under different small perturbations. These perturbations can be introduced either directly to the input images or to the intermediate features extracted from the model. These two approaches are commonly referred to as image perturbation and feature perturbation, respectively.

As shown in Figure 2, for each labeled batch, the detailed training process is described in Section 2.2.1. On the other hand, for each unlabeled batch, we perform three independent data augmentations on the data, resulting in three augmented versions: $x^{w}$, $x^{s_{1}}$, and $x^{s_{2}}$, where $x^{w}$ is obtained through weak augmentation such as cropping, and $x^{s_{2}}$ is obtained through strong augmentation such as color jitter. The subsequent forward propagation process includes three forward flows, which are (1) The simplest flow: $x^{w}\to f\to p^{w}$, (2) Image-level strong perturbation flow: $x^{s_{1}}$, $x^{s_{2}}\to f\to p^{s_{1}}$, $p^{s_{2}}$, and (3) Feature perturbation flow: $x^{w}\to g\to P\to h\to p^{fp}$. The outputs of (1) and (2) are collectively referred to as unified perturbations, and must remain consistent. Similarly, the outputs of (1) and (3) are referred to as dual-stream perturbations, and must also remain consistent. Thus, the total loss function $L_{u}$ can be expressed as:

(3) $$\begin{array}{c} L_{u}=\frac{1}{B_{u}}\sum I\left(\max\left(p^{w}\right)\geq \tau \right)\cdot \\ \;\left(\lambda H\left(p^{w},p^{fp}\right)+\frac{\mu }{2}\left(H\left(p^{w},p^{s_{1}}\right)+H\left(p^{w},p^{s_{2}}\right)\right)\right), \end{array}$$

where $B_{u}$ represents the batch size for unlabeled data, and τ is a predefined confidence threshold used to filter out noisy labels. H minimizes the entropy between two input probability distributions.

#### 2.2.3. Training Stage 2: Training of Multi-Scale Fusion Modules

Because our trained model requires inputs of size 1024×1024, when applying it to WSI inference, the WSI needs to be divided into patches of size 1024×1024 for processing. Considering the high resolution of the WSI, our model cannot send the entire WSI into the network at once during inference, so it needs to be divided into smaller patches and inferred individually, and then the results are combined to form the overall inference result of the WSI. However, this method causes the model to lose the context between patches during inference, resulting in discontinuity in the inference results, especially at the boundaries between blocks. In order to solve this problem, we propose a multi-scale input model based on the initial segmentation network, which fully considers the surrounding information of the patches to ensure more accurate segmentation in WSIs.

Compared to the initial segmentation model, the multi-scale model incorporates two attentional feature fusion (AFF) modules [35]. The model takes an image of size 4096×4096 as input and generates the corresponding segmentation result for the region cropped from the central 2048×2048 portion of the input, with a size of 256×256. The process of the multi-scale model is illustrated in Figure 2. The input data is processed in three distinct ways:

1. Cropped in the four corners according to the size of 2048×2048;
2. Cropped in the center according to the size of 2048×2048;
3. Scaled to the size of 2048×2048.

After processing, they are individually passed through the PIDNet with frozen weights, resulting in three different outputs. The top branch output is remapped as the large image, with the cropped center referred to as $p_{1}$. The middle branch output remains unprocessed and is denoted as $p_{2}$. The bottom branch output is cropped in the center and labeled as $p_{3}$. These three outputs are fused using two AFF modules to obtain final output *p*:

(4) $$p=\mathrm{AFF}\left(\mathrm{AFF}\left(p1,p2\right),p3\right),$$

where the specific order of operations, from $p_{1}$ to $p_{3}$, was chosen based on the hierarchical nature of the data and the desired behavior of the model. This sequential approach allows the model to capture and integrate information at different levels of detail, starting from the finer details and gradually incorporating the broader context. The fusion of the output *p* is then compared with the ground truth to calculate the cross-entropy loss, which is used to train the two AFF modules. It is important to note that PIDNet does not participate in this training stage.

## 3. Results

### 3.1. Quantitatively Experiments for Segmentation Task

We conducted a thorough comparison of our proposed method with various segmentation models on the test set. For all experiments, we utilized the stochastic gradient descent (SGD) optimizer with the following parameters: learning rate of 0.001, momentum of 0.9, and weight decay of 0.0005. Each model was trained for 300 epochs. To ensure the statistical stability of the results, we employed a five-fold cross-validation approach. The results are presented in Table 1. Notably, our proposed method achieved impressive scores of 84.16% and 76.33% for $IoU_{dcis}$ and $IoU_{ic}$, respectively, surpassing the performance of other segmentation models. This demonstrates its robust capability in accurately segmenting tumor regions.

Furthermore, we investigated the impact of incorporating semi-supervised learning and multi-scale fusion modules on model performance. The IoU scores on the test set for different method strategies are shown in Table 2. Obviously, when compared to other ablated variants, our proposed method achieved the highest mean IoU on the test set, with an improvement of 1.40% over the baseline model. Additionally, the proposed method outperformed using only semi-supervised learning and only multi-scale strategies by 0.61% and 0.89%, respectively. These comprehensive research findings provide compelling evidence for the effectiveness of both the semi-supervised learning strategy and the multi-scale strategy in improving model segmentation performance.

**Figure 3:**
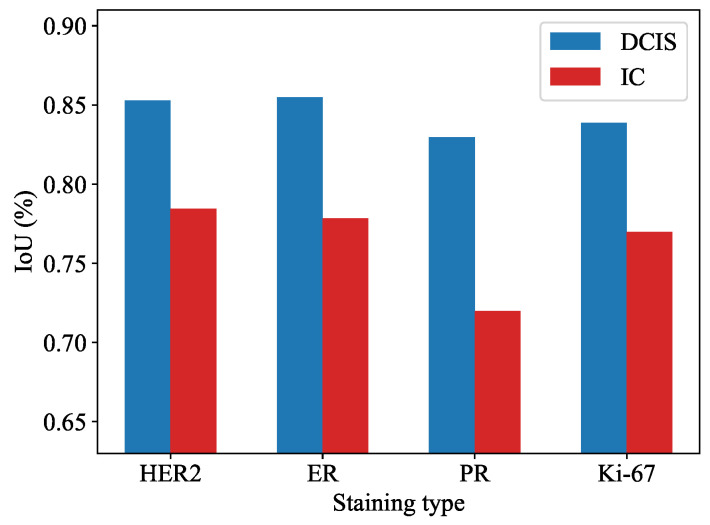
Performance of the proposed method on the test set based on tumor type (DCIS or IC) and staining type (HER2, ER, PR, Ki-67).

The performance of the proposed method was examined on the test set, considering tumor type and staining type. Figure 3 illustrates that the IoU score was highest for HER2 staining images, potentially due to the membrane staining, which aids in improved recognition of tumor borders. With the exception of a slightly lower segmentation performance in PR staining images for invasive carcinoma compared to other immunohistochemical staining types, the segmentation performance was generally comparable across different staining types.

### 3.2. Visual Analysis

**Figure 4:**
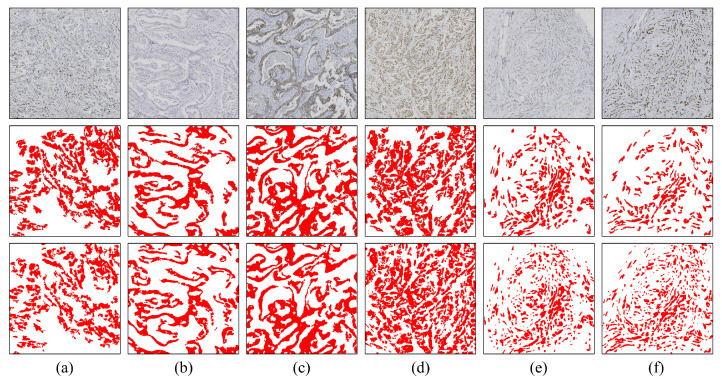
Segmentation results of pure invasive carcinoma areas under various staining types. (**a**) Ki-67, 15%; (**b**) ER, negative; (**c**) HER2, 2+; (**d**) ER, positive; (**e**) HER2, 1+; (**f**) PR, positive. Rows 1–3 represent images, model predictions, and ground truth, respectively.

In the visual analysis section, we present a comparison between the segmentation results of the pure invasive carcinoma region and the ground truth provided by pathologists, as illustrated in Figure 4. In Figure 4, we observe the segmentation results of pure invasive carcinoma areas with various staining types, revealing a high heterogeneity in terms of nuclear morphology, tissue structure, and staining intensity of the epithelial tissue areas. Nonetheless, the model exhibits overall good stability and accuracy and can accurately segment invasive carcinoma in these areas. However, in cases where the nests of invasive carcinoma are very small, as shown in Figure 4e,f, the segmentation performance of the model is somewhat reduced due to the small area of the cancer nests and reduced discriminability.

**Figure 5:**
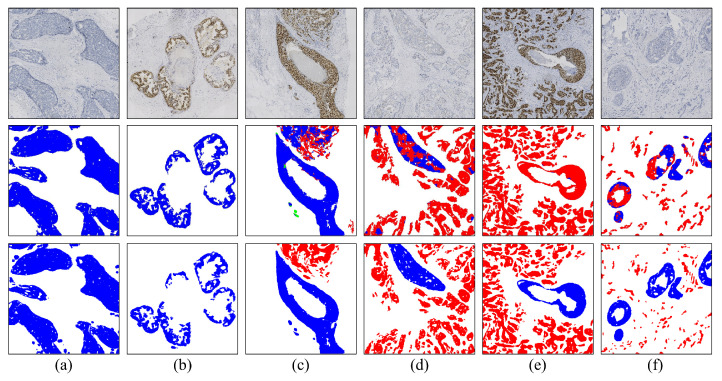
Segmentation results of pure DCIS areas (**a**,**b**) and areas with a mixture of DCIS and invasive carcinoma (**c**–**f**) under various staining types. Rows 1–3 represent images, model predictions, and ground truth, respectively.

Furthermore, we present the segmentation results in both the pure DCIS region and the region with a mixture of DCIS and invasive carcinoma, as shown in Figure 5. The segmentation results for pure DCIS areas (Figure 5a,b) closely follow the ground truth provided by pathologists. Comparing the segmentation results of the invasive carcinoma region in Figure 4, we find that the IoU index of the pure DCIS region is relatively higher than that of the pure invasive carcinoma region, mainly due to the larger size of the cancer nests in DCIS. However, for areas with a mixture of DCIS and invasive carcinoma (Figure 5c–f), the model’s segmentation performance is notably worse than when only a single component exists in the field of view. In some cases, the model struggles to recognize DCIS (Figure 5e). Generally, negative-staining samples show slightly better segmentation performance than positive-staining samples, possibly because high staining intensity in positive-staining samples overwhelms the texture feature, making it challenging for the model to distinguish between DCIS and invasive carcinoma in such areas.

**Figure 6:**
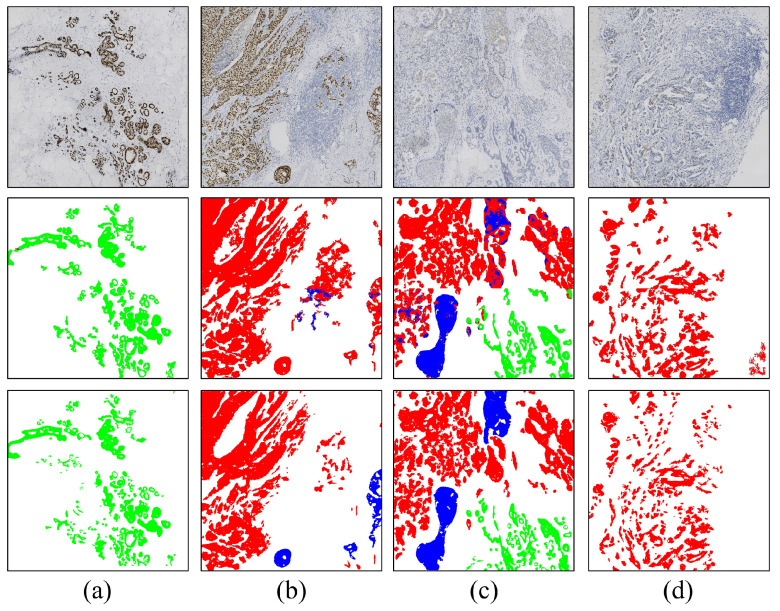
Segmentation results from some special cases. (**a**) Pure lobular area; (**b**) area with a mixture of DCIS and invasive carcinoma with lymphocytic infiltration; (**c**) area with a mixture of DCIS and invasive carcinoma with lobular; (**d**) invasive carcinoma area with lymphocytic infiltration. Rows 1–3 represent images, model predictions, and ground truth, respectively.

In addition, Figure 6 displays some other situations, including the presence of normal lobules (Figure 6a,c) and lymphocyte areas (Figure 6b,d). The model can effectively distinguish between normal lobules and tumor regions, but its performance is less stable when dealing with lymphocyte aggregation. One reason might be that the model tends to classify the lymphocyte-enriched region as the background class. Segmenting it as a separate category could potentially improve the performance. Overall, the model exhibits a certain level of discriminability for distinguishing invasive and non-invasive carcinoma regions, thereby avoiding the inclusion of normal epithelial cells and lymphocytes during the process of quantitative biomarker analysis.

### 3.3. Role of Invasive Carcinoma Mask in Ki-67 Quantification

**Figure 7:**
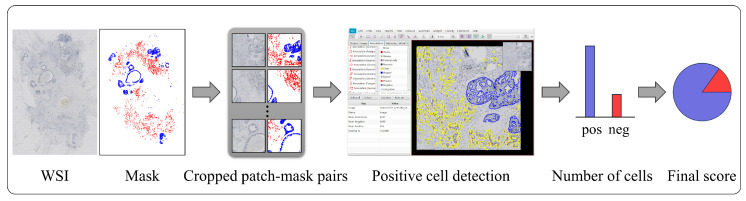
Processing steps for WSI-level Ki-67 quantification.

To better illustrate the role of the proposed invasive carcinoma segmentation model in immunohistochemical quantification, we selected 29 cases of Ki-67-stained WSIs with IBC-NST, where the DCIS constituted more than 10% of the total tumor area. Using our segmentation model, we accurately obtained the masks of DCIS and invasive carcinoma areas in these cases using our trained model. Subsequently, we employed the QuPath software to quantitatively calculate the Ki-67 indices of the WSIs based on the obtained masks. An example of the processing steps for a single case is presented in Figure 7.

**Figure 8:**
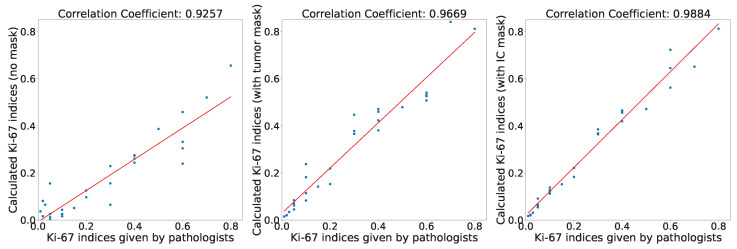
Correlation plots of Ki-67 indices under different conditions.

**Figure 9:**
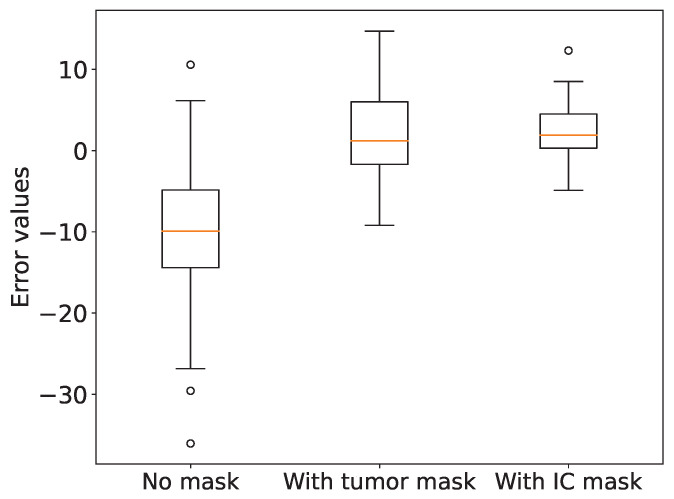
Box plot of Ki-67 index errors for different tumor area masks.

For comparative analysis, we calculated the Ki-67 indices under three conditions: without any mask, with a tumor area mask, and with an invasive carcinoma area mask. We then compared these indices with the Ki-67 indices provided by pathologists. The corresponding scatter plots are illustrated in Figure 8. It is evident that the Ki-67 indices calculated based on the invasive carcinoma area mask exhibit the highest correlation coefficient with the pathologists’ Ki-67 indices, with a value of 0.9884. To evaluate the accuracy of the calculated Ki-67 indices, we generated a box plot (Figure 9) presenting the Ki-67 index errors. The mean Ki-67 index errors for no mask, tumor area mask, and invasive carcinoma area mask were -10.19, 1.78, and 2.46, respectively, while their corresponding standard deviations were 10.17, 6.03, and 3.69. We observed that the lower Ki-67 index calculated without a mask is mainly attributed to areas with a significant presence of immune lymphocytes, which often yield negative results, leading to considerable errors. However, upon incorporating either the tumor area mask or the invasive carcinoma area mask, the accuracy of the Ki-67 quantification improved significantly. Although the calculation with the invasive carcinoma mask resulted in slightly higher error than the tumor area mask, its standard deviation was smaller, indicating more stable quantitative scores. Consequently, in practical applications, the utilization of the invasive carcinoma area mask can lead to more reliable and consistent results.

## 4. Discussion

In this study, we developed a deep learning algorithm for segmenting invasive carcinoma regions in IHC-stained WSI. The algorithm achieves an average IoU of 80.16% for DCIS and invasive carcinoma segmentation. The correlation coefficient between the Ki-67 scores calculated based on the invasive carcinoma masks obtained by our model and the scores provided by pathologists is as high as 0.9884. Detailed visual results enhance interpretability and practical application. As a powerful assistant for pathologists, our algorithm lays the foundation for accurate IHC scoring.

In recent years, there has been a significant body of work concerning the quantitative analysis of IHC-stained slides. Some studies [20,22,23,27] have focused on the detection or segmentation of tumor-positive and tumor-negative cells, yet they have not taken into account the automatic segmentation of IC regions. Yao et al. [26] have approached IHC quantification by calculating grayscale features for each tile and generating WSI-level feature maps based on these characteristics, subsequently using these maps for HER2 grading. However, by not separately computing for IC regions, this method may be influenced by non-tumor areas or DCIS regions, potentially leading to biased results. Qaiser at el. [25] have introduced a deep reinforcement learning approach for the automatic HER2 scoring. Unlike fully supervised models that process all areas of a given input image, the proposed model treats IHC scoring as a sequential selection task, effectively locating diagnostically relevant areas by deciding on viewing positions. Although the proposed model has the potential to address other histological image analysis challenges where precise pixel-level annotations are hard to obtain, it risks missing crucial tiles with decisive features, as it does not globally consider the expression of immunohistochemical markers in tumor regions as a pathologist would. Valkonen et al. [24] developed a deep-learning-based digital mask for automatic epithelial cell detection using dual-stained fluorescent cytokeratin-Ki-67 and consecutive H&E-IHC staining as training material. While this method enables the model to effectively segment tumor regions in IHC, it similarly fails to exclude in situ carcinoma regions. Feng et al. [21] have achieved fully automatic Ki-67 quantification using H&E-stained images. They first identified DCIS regions in H&E, and then mapped these identified regions onto IHC images through rigid registration. However, this method demands high accuracy from the registration algorithm. In practical scenarios, H&E and IHC slides may not be adjacent, and small tumor regions can be challenging to match one-to-one, leading to potential inaccuracies due to rigid registration.

To sum up, existing methods have their limitations, notably the failure to consider DCIS regions, hindering fully automated IHC quantification. To overcome this, we have proposed, for the first time, a method that directly identifies DCIS and IC within IHC images, bringing fully automated IHC quantification one step closer to clinical application.

However, this study still had some limitations. Firstly, the model’s segmentation consistency across WSIs is currently suboptimal. The algorithm’s accuracy and reliability are compromised by its limited ability to integrate and analyze data from an entire WSI or multiple stained WSIs from the same case. Secondly, the model’s training has been conducted on a relatively homogenous set of samples, and it is restricted to a narrow range of classes it can segment. Lastly, the absence of a deep-learning-based nuclei detection algorithm means that the current model lacks the capacity for end-to-end automatic evaluation of IHC quantification scores for entire WSIs.

Regarding future improvements, our research aims to enhance the overall segmentation consistency of the model on WSIs by incorporating results from an entire WSI or multiple stained WSIs from each case. This integration will lead to improved accuracy and reliability of the algorithm. Additionally, we intend to train the model with more diverse samples and expand the number of classes it can effectively segment. This expansion will enable the model to handle more complex scenarios, including identifying micro-invasive regions, accurately segmenting regions of multiple categories from the same field of view., considering additional tissue types such as atypical ductal hyperplasia (ADH) and usual ductal hyperplasia (UDH), and addressing atypical cases, such as samples from patients undergoing neoadjuvant chemotherapy. These advancements are expected to widen the applicability and effectiveness of our model in practical clinical settings. Furthermore, we plan to employ a deep-learning-based nuclei detection algorithm. This addition will enable the model to achieve end-to-end automatic evaluation of immunohistochemistry quantification scores for an entire WSI.

## 5. Conclusions

This study proposes a deep-learning algorithm for segmenting invasive carcinoma regions in breast cancer IHC-stained WSIs. The algorithm achieves promising results in the segmentation task. Ki-67 quantification results based on segmented invasive carcinoma masks demonstrates high consistency with pathologists’ assessments. It provides valuable assistance in clinical settings, improving breast cancer diagnosis and treatment efficiency. Future developments will strengthen its practical application in pathological diagnosis, enhancing usability across various clinical scenarios.

## Figures and Tables

**Table 1:** Comparison of proposed method with various segmentation models on the test set.

Method	IoU (DCIS, %)	IoU (IC, %)	Average
ResNet50 [36] + Unet [37]	73.83±0.70	67.95±1.11	70.89±0.62
Mit-b5 [38] + Unet	80.98±2.90	65.72±3.86	73.35±2.59
MagNet [39]	78.44±0.54	70.72±0.14	74.58±0.22
FCtL [40]	80.66±2.45	66.97±1.07	73.81±0.23
PIDNet	81.33±3.47	76.37±1.42	78.85±1.57
Proposed	84.16±2.72	76.33±3.46	80.25±1.26

**Table 2:** Ablation study of semi-supervised learning and multi-scale fusion modules.

Method	IoU (DCIS, %)	IoU (IC, %)	Average
PIDNet	81.33±3.47	76.37±1.42	78.85±1.57
PIDNet + Unimatch	81.58±2.87	77.70±2.58	79.64±0.81
PIDNet + AFF	81.62±1.07	77.10±3.12	79.36±0.75
Proposed	84.16±2.72	76.33±3.46	80.25±1.26

## Data Availability

The datasets used during the current study are available from the corresponding author on reasonable request.

## Abbreviations

The following abbreviations are used in this manuscript:

ADB	auxiliary derivative branch
ADH	atypical ductal hyperplasia
AFF	attentional feature fusion
DCIS	ductal carcinoma in situ
ER	estrogen receptor
HER2	human epidermal growth factor receptor 2
IBC-NST	invasive breast carcinoma of no special type
IC	invasive carcinoma
IHC	immunohistochemistry
PR	progesterone receptor
ROI	region of Interest
SGD	stochastic gradient descent
UDH	usual ductal hyperplasia
WSI	whole-slide image

## References

[1] Chhikara B.S., Parang K. (2023). Global Cancer Statistics 2022: The trends projection analysis. Chem. Biol. Lett..

[2] WHO (2019). WHO Classification of Tumors–Breast Tumors.

[3] Zhang L., Huang Y., Feng Z., Wang X., Li H., Song F., Liu L., Li J., Zheng H., Wang P. (2019). Comparison of breast cancer risk factors among molecular subtypes: A case-only study. Cancer Med..

[4] Zaha D.C. (2014). Significance of immunohistochemistry in breast cancer. World J. Clin. Oncol..

[5] Dabbs D.J. (2021). Diagnostic Immunohistochemistry E-Book: Theranostic and Genomic Applications.

[6] Mathew T., Niyas S., Johnpaul C., Kini J.R., Rajan J. (2022). A novel deep classifier framework for automated molecular subtyping of breast carcinoma using immunohistochemistry image analysis. Biomed. Signal Process. Control..

[7] López C., Lejeune M., Salvadó M.T., Escrivà P., Bosch R., Pons L.E., Álvaro T., Roig J., Cugat X., Baucells J. (2008). Automated quantification of nuclear immunohistochemical markers with different complexity. Histochem. Cell Biol..

[8] Qaiser T., Mukherjee A., Reddy Pb C., Munugoti S.D., Tallam V., Pitkäaho T., Lehtimäki T., Naughton T., Berseth M., Pedraza A. (2018). Her 2 challenge contest: A detailed assessment of automated her 2 scoring algorithms in whole slide images of breast cancer tissues. Histopathology.

[9] Gavrielides M.A., Gallas B.D., Lenz P., Badano A., Hewitt S.M. (2011). Observer variability in the interpretation of HER2/neu immunohistochemical expression with unaided and computer-aided digital microscopy. Arch. Pathol. Lab. Med..

[10] Chung Y.R., Jang M.H., Park S.Y., Gong G., Jung W.H. (2016). Interobserver variability of Ki-67 measurement in breast cancer. J. Pathol. Transl. Med..

[11] Leung S.C., Nielsen T.O., Zabaglo L.A., Arun I., Badve S.S., Bane A.L., Bartlett J.M., Borgquist S., Chang M.C., Dodson A. (2019). Analytical validation of a standardised scoring protocol for Ki67 immunohistochemistry on breast cancer excision whole sections: An international multicentre collaboration. Histopathology.

[12] Cai L., Yan K., Bu H., Yue M., Dong P., Wang X., Li L., Tian K., Shen H., Zhang J. (2021). Improving Ki67 assessment concordance by the use of an artificial intelligence-empowered microscope: A multi-institutional ring study. Histopathology.

[13] Fisher N.C., Loughrey M.B., Coleman H.G., Gelbard M.D., Bankhead P., Dunne P.D. (2022). Development of a semi-automated method for tumour budding assessment in colorectal cancer and comparison with manual methods. Histopathology.

[14] Hondelink L.M., Hüyük M., Postmus P.E., Smit V.T., Blom S., von der Thüsen J.H., Cohen D. (2022). Development and validation of a supervised deep learning algorithm for automated whole-slide programmed death-ligand 1 tumour proportion score assessment in non-small cell lung cancer. Histopathology.

[15] Ba W., Wang S., Shang M., Zhang Z., Wu H., Yu C., Xing R., Wang W., Wang L., Liu C. (2022). Assessment of deep learning assistance for the pathological diagnosis of gastric cancer. Mod. Pathol..

[16] Song Z., Zou S., Zhou W., Huang Y., Shao L., Yuan J., Gou X., Jin W., Wang Z., Chen X. (2020). Clinically applicable histopathological diagnosis system for gastric cancer detection using deep learning. Nat. Commun..

[17] Van der Laak J., Litjens G., Ciompi F. (2021). Deep learning in histopathology: The path to the clinic. Nat. Med..

[18] Ho C., Zhao Z., Chen X.F., Sauer J., Saraf S.A., Jialdasani R., Taghipour K., Sathe A., Khor L.Y., Lim K.H. (2022). A promising deep learning-assistive algorithm for histopathological screening of colorectal cancer. Sci. Rep..

[19] Niazi M.K.K., Parwani A.V., Gurcan M.N. (2019). Digital pathology and artificial intelligence. Lancet Oncol..

[20] Geread R.S., Sivanandarajah A., Brouwer E.R., Wood G.A., Androutsos D., Faragalla H., Khademi A. (2020). Pinet—An automated proliferation index calculator framework for Ki67 breast cancer images. Cancers.

[21] Feng M., Deng Y., Yang L., Jing Q., Zhang Z., Xu L., Wei X., Zhou Y., Wu D., Xiang F. (2020). Automated quantitative analysis of Ki-67 staining and HE images recognition and registration based on whole tissue sections in breast carcinoma. Diagn. Pathol..

[22] Huang Z., Ding Y., Song G., Wang L., Geng R., He H., Du S., Liu X., Tian Y., Liang Y. (2020). Bcdata: A large-scale dataset and benchmark for cell detection and counting. Proceedings of the Medical Image Computing and Computer Assisted Intervention 23rd International Conference (MICCAI 2020).

[23] Negahbani F., Sabzi R., Pakniyat Jahromi B., Firouzabadi D., Movahedi F., Kohandel Shirazi M., Majidi S., Dehghanian A. (2021). PathoNet introduced as a deep neural network backend for evaluation of Ki-67 and tumor-infiltrating lymphocytes in breast cancer. Sci. Rep..

[24] Valkonen M., Isola J., Ylinen O., Muhonen V., Saxlin A., Tolonen T., Nykter M., Ruusuvuori P. (2019). Cytokeratin-supervised deep learning for automatic recognition of epithelial cells in breast cancers stained for ER, PR, and Ki-67. IEEE Trans. Med. Imaging.

[25] Qaiser T., Rajpoot N.M. (2019). Learning where to see: A novel attention model for automated immunohistochemical scoring. IEEE Trans. Med. Imaging.

[26] Yao Q., Hou W., Wu K., Bai Y., Long M., Diao X., Jia L., Niu D., Li X. (2022). Using Whole Slide Gray Value Map to Predict HER2 Expression and FISH Status in Breast Cancer. Cancers.

[27] Priego-Torres B.M., Lobato-Delgado B., Atienza-Cuevas L., Sanchez-Morillo D. (2022). Deep learning-based instance segmentation for the precise automated quantification of digital breast cancer immunohistochemistry images. Expert Syst. Appl..

[28] Huang J., Mei L., Long M., Liu Y., Sun W., Li X., Shen H., Zhou F., Ruan X., Wang D. (2022). Bm-net: Cnn-based mobilenet-v3 and bilinear structure for breast cancer detection in whole slide images. Bioengineering.

[29] Van Rijthoven M., Balkenhol M., Siliņa K., Van Der Laak J., Ciompi F. (2021). HookNet: Multi-resolution convolutional neural networks for semantic segmentation in histopathology whole-slide images. Med. Image Anal..

[30] Ni H., Liu H., Wang K., Wang X., Zhou X., Qian Y. (2019). WSI-Net: Branch-based and hierarchy-aware network for segmentation and classification of breast histopathological whole-slide images. Proceedings of the Machine Learning in Medical Imaging: 10th International Workshop, Held in Conjunction with MICCAI 2019 (MLMI 2019).

[31] Bankhead P., Loughrey M.B., Fernández J.A., Dombrowski Y., McArt D.G., Dunne P.D., McQuaid S., Gray R.T., Murray L.J., Coleman H.G. (2017). QuPath: Open source software for digital pathology image analysis. Sci. Rep..

[32] Xu J., Xiong Z., Bhattacharyya S.P. PIDNet: A Real-Time Semantic Segmentation Network Inspired by PID Controllers. Proceedings of the IEEE/CVF Conference on Computer Vision and Pattern Recognition.

[33] Takikawa T., Acuna D., Jampani V., Fidler S. Gated-scnn: Gated shape cnns for semantic segmentation. Proceedings of the IEEE/CVF International Conference on Computer Vision.

[34] Yang L., Qi L., Feng L., Zhang W., Shi Y. Revisiting weak-to-strong consistency in semi-supervised semantic segmentation. Proceedings of the IEEE/CVF Conference on Computer Vision and Pattern Recognition.

[35] Dai Y., Gieseke F., Oehmcke S., Wu Y., Barnard K. Attentional feature fusion. Proceedings of the IEEE/CVF Winter Conference on Applications of Computer Vision.

[36] He K., Zhang X., Ren S., Sun J. Deep residual learning for image recognition. Proceedings of the IEEE Conference on Computer Vision and Pattern Recognition.

[37] Ronneberger O., Fischer P., Brox T. (2015). U-net: Convolutional networks for biomedical image segmentation. Proceedings of the Medical Image Computing and Computer-Assisted Intervention: 18th International Conference (MICCAI 2015).

[38] Xie E., Wang W., Yu Z., Anandkumar A., Alvarez J.M., Luo P. (2021). SegFormer: Simple and efficient design for semantic segmentation with transformers. Adv. Neural Inf. Process. Syst..

[39] Huynh C., Tran A.T., Luu K., Hoai M. Progressive semantic segmentation. Proceedings of the IEEE/CVF Conference on Computer Vision and Pattern Recognition.

[40] Li Q., Yang W., Liu W., Yu Y., He S. From contexts to locality: Ultra-high resolution image segmentation via locality-aware contextual correlation. Proceedings of the IEEE/CVF International Conference on Computer Vision.

